# Commentary on: A study of genotype, mutants and nucleotide sequence of HBV in Pakistan: Genotype, mutants, and nucleotide sequence of HBV

**Published:** 2011-04-01

**Authors:** Seyed Mohammad Jazayeri

**Affiliations:** 1 Hepatitis B Molecular Laboratory, Department of Virology, School of Public Health, Tehran University of Medical Sciences, Tehran, IR Iran

**Keywords:** Genotype, Hepatitis B virus, Epidemiology Mutants

Dear Editor,

I read with interest the article "A study of genotype, mutants and nucleotide sequence of HBV in Pakistan" by Mumtaz et al. [1] in your journal. The study revealed a prevalence of 96.2% of genotype D in different areas of Pakistan. Hepatitis B virus (HBV) has been classified into 10 genotypes (based on the intergroup divergence of > 8% to < 17%), with a characteristic geographic distribution that largely coincides with human history and migration. Among the different HBV genotypes, genotype D has been found worldwide, with its highest prevalence in the Mediterranean, the Middle East, and Southern Asia [2]. Mumtaz et al. stated that they wanted to determine the country-wide distribution of HBV genotypes in Pakistan. However, comparing the previous data from this country using larger sample sizes showed conflicting results. We collected all the available data regarding the prevalence of HBV genotypes in Pakistan population and we found somewhat contradictory results on the type and prevalence of different HBV genotypes circulating in this region (Table 1). The majority of studies carried out on the determination of HBV genotypes utilized a nested-multiplex PCR using genotype-specific primers. The rates of genotype D in these studies ranged between 13% and 70% (Table 1). However, authors who used an INNO-Lipa methodology found prevalence rates between 96.2% and 100% (Table 1). The genotype prevalence was found to be different in the Sind and Punjab provinces in three reports (Table 1). We consider that these discrepancies may be explained in part by the selection of patients as well as the differences in the sensitivity of the assays used to detect signature sequences within the isolates.

Moreover, Mumtaz et al. randomly selected 3 samples, followed sequencing in a phylogenetic analysis, and stated that they compared the nucleotide sequences of isolates from Pakistan to isolates from the rest of the world. We think that this type of selection imposes a bias on the phylogeny results. First, despite being a gold standard for methodology, direct sequencing does not detect the viral mixture pool (quasispecies). Second, how do these 3 sequences reflect the dominant sequences from Pakistan with such a large number of diversifications and recombinations (Table 1)? We think that a higher number of sequencing data with a higher number of sequencing data with fewer mutations. should have been employed for more precise comparisons and to eliminate the noise of the software on the processing data. Also, the authors used genotype G for outgrouping; however, they should have used genotype E for this purpose because genotype E is more precise for outgrouping of suspected genotype D isolates (Norder, personal communication, April 25, 2010).

The evolution of HBV genotypes in the world (especially the widespread genotype D in Southern Asia) is confusing. Summarizing the reports from Pakistan, we found an average prevalence of genotypes D (62%), A (14%), C (6%), other genotypes, including B (4%), and recombinants (10%) [3]. Furthermore, the E and F genotypes [4] are unusual in the Pakistan population. These unusual genotypes deserve further investigation. This diversity of genotypes and the high rate of recombination (10%) between different genotypes in Pakistan indicate a high rate of intermixing among infected populations, perhaps driven by the T-cell selection in this distribution, reflected by the divergence of amino-acid substitutions in the signature sequences of the HBV genome. We propose that the HBV-infected ancestors of Caucasians acquired genotype D, then migrated in 3 directions: one group moved west towards Europe, another group moved south to Persia, and the last group migrated to India. People infected with type D of the virus before migrating then transmitted the virus generation by generation after migration. This is why the dominant genotype in southern Asia (including India, Iran, and Pakistan) and most parts of Europe is D [3]. Based on molecular epidemiology studies, knowing the HBV genotypic distribution in Pakistan's population is of importance because it has been shown that they influence the clinical prognosis, response to therapy and, the transmission status of the disease.

**Table 1:** Prevalence of HBV genotypes in Pakistan's population

** Author **	** Samples **(No.)	** Location **(Predomiant Genotype)	** Genotype D **	** Genotype A **	** Method **
** Baig S et al. **(2008) (5)	201	-	128 (64%)	47 (23%)	Type-specific primer
** Baig S et al. **(2009) (6)	129	-	98 (76%)	24 (18.6%)	Type-specific primer
** Baig S et al. **(2009) (7)	315	Sind	219 (70%)	65(20%)	Type-specific primer
** Baig S et al. **(2007) (8)	295	-	208 (70%)	59 (20%)	Type-specific primer
** Alam MM et al. **(2007) (9)	110	-	66 (65.3%)	5 (4.9%)	Type-specific primer
** Alam MM et al. **(2007) (10)	56	NWFP	35 (62.5%)	15 (8.9%)	Type-specific primer
** Awan Z et al. **(2010) (4)	269	Sind (A) /Punjab (C)/Baloochistan (B) /Kheybar (C)	39 (13%)	43 (14.3%)	Type-specific primer
** Noorali S et al. **(2008) (12)	180	Sind	150 (83.3%)	0	Type-specific primer Sequencing
** Noorali S et al. **(2008) (13)	180	Sind	151 (83.8%)	0	PCR
** Abbas Z et al. **(2006) (14)	109	Sind	109 (100%)	0	INNO-Lipa
** Mumtaz K et al. **(2011) (1)	257	Sind/Punjab/Baloochestan/NWFP	247 (96%)	1	INNO-Lipa
** Ahmed CS et al. **(2009) (15)	236	Sind (D)/Punjab (D)	220 (93.2%)	2 (0.8%)	RFLP-Sequencing
